# Clinical and cost-effectiveness of collaborative traditional Korean and Western medicine treatment for low back pain: A protocol for a prospective observational exploratory study

**DOI:** 10.1097/MD.0000000000012595

**Published:** 2018-09-28

**Authors:** Eun Hye Hyun, Hye-Yoon Lee, Hye Won Kim, Hyun Min Kim, Eun Jung Kim, Seon Jong Kim, Yoon Gyung Song, Young Il Kim, Woo Jin Nam, Dong Hyung Seo, Sang Ho Lee, NamKwen Kim

**Affiliations:** a School of Korean Medicine, Pusan National University; b National Clinical Research Center for Korean Medicine, Pusan National University Korean Medicine Hospital, Yangsan-si, Gyeongsangnam-do; c Dongguk University Bundang Oriental Hospital, Seongnam-si, Gyeonggi-do; d Dongshin University Mokpo Oriental Hospital, Mokpo-si, Jeollanam-do; e Gachon University Gil Korean Medicine Hospital, Jung-gu, Incheon; f Daejeon University, Dunsan Korean Medicine Hospital, Seo-gu, Daejeon; g Samse Korean Medical Hospital, Geumjeong-gu, Busan; h Design Hospital, Jeonju-si, Jeollabuk-do; i Mokhuri Neck and Back Hospital, Gangdong-gu, Seoul, Republic of Korea.

**Keywords:** collaborative treatment, low back pain, prospective, traditional Korean medicine observational study

## Abstract

**Background:**

In South Korea, a few patients with low back pain (LBP) are currently being treated with a combination of traditional Korean medicine (KM) and Western medicine (WM). Although a recent research has reported results regarding patient satisfaction and exploratory effectiveness, evidence of comparative effectiveness still needs to be reviewed. The aim of this study is to evaluate the clinical and cost-effectiveness of KM and WM collaborative treatment (CT) compared with that of sole treatment (ST) for patients with LBP in Korea.

**Method/design:**

This multisite, prospective observational comparative effectiveness research study is part of a nationwide pilot project for KM and WM collaboration launched by the Korean Ministry of Health and Welfare. The duration of the study is 8 weeks, and the target number of inclusion is 360 patients. Participants receive treatment according to their treatment plan, and a researcher conducts investigations thrice, every 4 weeks. In the final analysis, the merged data from the participants’ questionnaire responses, hospital medical records, and administrative data, and Health Insurance Review and Assessment service data will be compared between the CT and ST groups.

**Discussion:**

This study will provide clinical and economic information about CT for LBP, which might be a milestone for establishing future polices about this collaboration in Korea.

**Trial registration:**

The study protocol has been registered with the Clinical Research Information Service (KCT0002827).

## 1 Introduction

Low back pain (LBP) is reportedly one of the most burdensome ailments in South Korea, 2013.^[1]^ In addition, a study has shown that LBP affects approximately 540 million people worldwide,^[2]^ and another study revealed that the loss of working hours due to LBP has increased by 54% between 1990 and 2015.^[3]^

In Korea, a few patients with LBP use traditional Korean medicine (KM) and Western medicine (WM) collaboratively, known as a collaborative treatment (CT). This is a special treatment system in Korea, which has dual medical systems. In CT, a medical doctor (MD) and traditional Korean medical doctor (KMD) cooperate and conduct the examination and determine the diagnosis and treatment for the patient.^[4,5]^

Although a survey demonstrated that 66.1% of the general population in Korea were aware of CT and 89.4% intended to reuse it,^[6]^ continuous reviews regarding CT are still required. The positive aspects of CT include increased patient satisfaction and diversity of treatment options.^[7–9]^ However, its limitations include a conflict between an MD and KMD due to lack of academic understanding, a concern about medical service overuse, and shortcomings of the medical fee system.^[10–13]^

The National Health Insurance System (NHIS) of Korea covers a prior medical fee only if a patient receives treatment from both an MD and a KMD for the same disease on the same day.^[14]^ For instance, if a patient is prescribed pain medication by an MD for LBP and then treated with acupuncture by a KMD for LBP on the same day, it is considered that the patient underwent duplicate treatments. Hence, the fee for the acupuncture treatment is not covered by the NHIS and can be an additional burden on the patient.

In addition, the consultant's fee, which is provided to medical institutions when CT is performed, is not reimbursed for outpatients but only counted for inpatients, 2 to 5 times per month.^[15]^ Therefore, the fee system for CT requires improvement for both patients and healthcare providers.^[16–18]^

Based on the above status, the Ministry of Health and Welfare had conducted a first-stage pilot project to investigate utilization rate and patient satisfaction with CT, applying NHIS cover for both WM and KM treatments for the same disease.^[19]^ It revealed high patient satisfaction and frequent ailments of CT.^[7]^

Adding the recognition of consultant fee, the second-stage pilot project is designed to evaluate the clinical and cost-effectiveness of CT.^[20]^ This pilot study is part of the Registry for Korean Medicine and Western Medicine Collaborative Treatment and evaluates the clinical and cost-effectiveness of CT for LBP, which is one of the frequent ailments treated by CT.

## 2 Methods

### 2.1 Study design and setting

**Figure 1:**
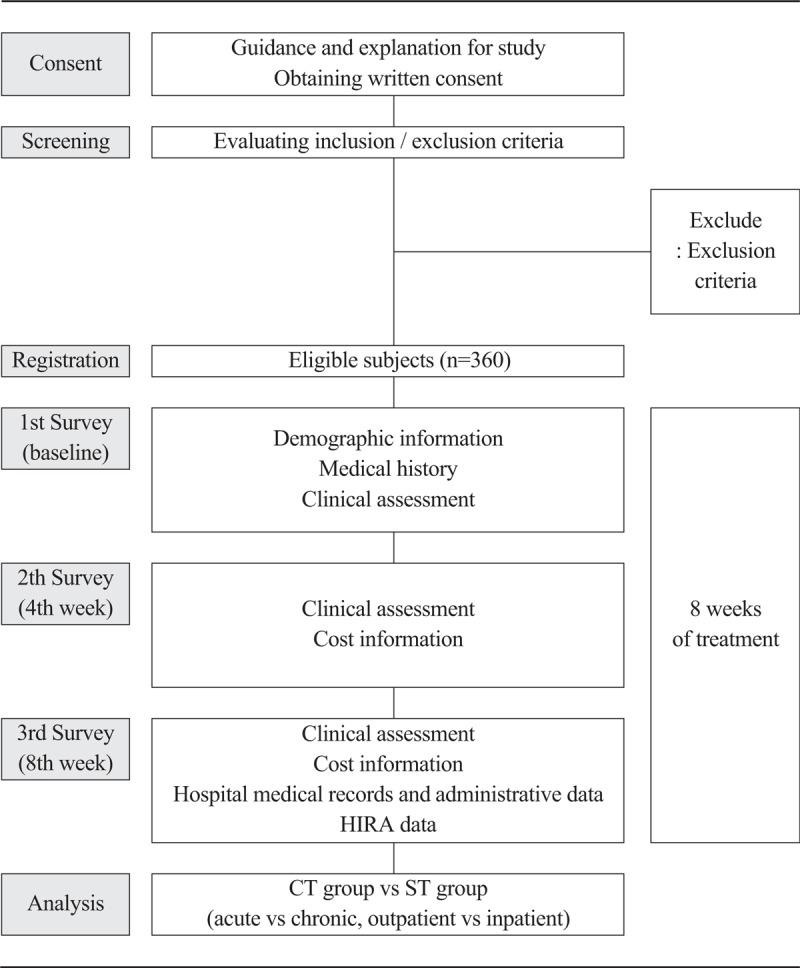
Flow chart of the study. CT = collaborative treatment, HIRA = Health Insurance Review and Assessment, ST = sole treatment.

Based on the International Society for Pharmacoeconomics and Outcomes Research's Good Research Practices dictates,^[21]^ the study is a multicenter and prospective observational comparative effectiveness research for the effectiveness of CT in patients with LBP. The patients with LBP are being recruited from 4 university-affiliated hospitals and 3 KM hospitals. A patient receives treatment according to their treatment plan. During or after the treatment, an 8-week study is conducted and a researcher assesses change in the patient's condition and cost of therapy thrice. In the final analysis, the merged data from the participants’ questionnaire responses, hospital medical records, and administrative data and Health Insurance Review and Assessment Service (HIRA) data will be compared between the CT and sole treatment (ST) groups. Figure 1 depicts the study design in a flow chart.

### 2.2 Participants

(1) Inclusion criteria
  1. Age ≥19 years and undergoing treatment at one of the institutions included in the study
  2. First-time visitor to the institution for LBP
  3. Voluntary informed consent
(2) Exclusion criteria
  1. Participation in another trial
  2. Difficulties or anticipated difficulties in complying with the study schedule
  3. Difficulties or anticipated difficulties in understanding and/or responding to study questionnaires
  4. Difficulties with study participation, as judged by the researchers

### 2.3 Participant consent and registration

A Patient who wishes to participate receives information regarding the study. Researchers explain to the participants that there is no relation between their participation and any provision of care, no obligation to participate, and no disadvantage if they choose not to participate or withdraw during the study course. After written informed consent is obtained, an MD or a KMD will judge the participant's eligibility, and those who meet the criteria are registered as study participants and enrollment numbers are assigned to anonymize them.

### 2.4 Source, measurement, and management

Data will be collected from the participants’ questionnaire responses, hospital medical records, and administrative data, and the HIRA data.

The participants’ questionnaire includes demographic information, medical history, clinical assessment, and cost information. Demographic information consists of birth date, sex, occupation, income, and any private medical insurance. Medical history consists of past treatment history for LBP, and other diseases or conditions apart from LBP. Clinical assessment consists of assessment indices for LBP. Cost information obtained from this questionnaire includes data that would not be obtainable from both hospital medical records and administrative data and HIRA data, such as medical expenses not covered by NHIS, supplementary foods, and auxiliary equipments for LBP.

Hospital medical records and administrative data include the number of visits, the number times the patient undergoes CT, the expenditure at the institution for LBP, the length of study participation, the department that requested CT, the department that accepted CT, and the date of initiation of CT.

The HIRA data are used for investigating the treatment modality (CT or ST) and the duration of LBP treatment. Moreover, medical expenses, such as the expenses covered by NHIS, can be obtained from the HIRA data.

All data will be entered and managed using iCReaT, which is an electronic-case report form designed by the Korea National Institute of Health. As executive agency of this study, the Monitoring Center for Korean Medicine and Western Medicine Collaboration is involved in data management by monitoring the study process. Table 1 shows the study data collection schedule.

**Table 1:**
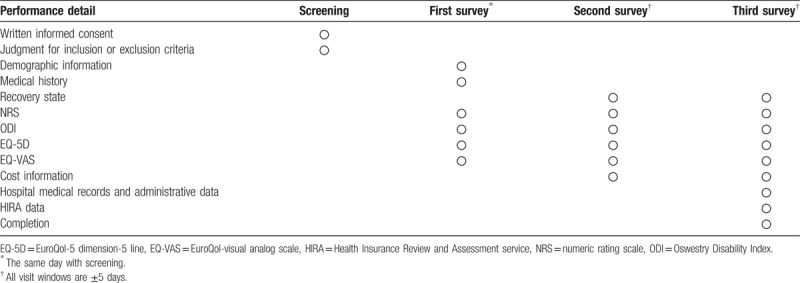
Data collection schedule.

### 2.5 Outcome measurement

#### 2.5.1 Primary outcome measurement

Primary outcome is the evaluation of clinical effectiveness assessed from the change in the numeric rating scale (NRS), Oswestry Disability Index (ODI), EuroQol-5 dimension-5 level (EQ-5D-5L), and EuroQol-visual analog scale (EQ-VAS) scores.

The NRS^[22]^ converts pain intensity to numerical values, using a horizontal line between 0 (no pain) and 10 (greatest pain severity). The ODI^[23,24]^ measures the degree of daily life disability caused by LBP. The EQ-5D-5L^[24,25]^ measures the quality of life using questions on mobility, self-care, activity, pain intensity, depression, and anxiety. The EQ-VAS^[25]^ scores quality of life using a horizontal line.

#### 2.5.2 Secondary outcome measurement

Secondary outcome is the evaluation of cost-effectiveness assessed from the cost per quality-adjusted life-years (QALYs), the incremental cost-effectiveness ratio (ICER), and the cost-effectiveness acceptability curve (CEAC).

The cost per QALYs is estimated based on the area under the curve method^[26]^ for each participant's quality of life data (e.g., the EQ-5D-5L and the EQ-VAS). The ICER represents the economic value of an intervention compared with an alternative. This is calculated by dividing the difference in total incremental cost by the difference in the chosen measure of health outcome or incremental effect.^[27]^ In CEAC, the horizontal axis represents the cost-effectiveness per ICER, and the vertical axis represents the probability of meeting that cost-effectiveness.^[28]^

### 2.6 Sample size rationale

The sample size was calculated by using the means (group 1 = 1.4, group 2 = 2.2) and standard deviations (sd 1 = 2.57, sd 2 = 2.76) from a previous study by NRS^[29]^ in the estimation equation^[30]^ via G-power program analysis (a 2-tailed, α = 0.05, β = 0.2). The result was 352, and the target number of inclusion was set at 360.

### 2.7 Statistical analyses

Outcomes will be compared between the main groups (CT and ST), which are stratified into 2 subgroups (acute or chronic^[31]^ and outpatient or inpatient^[32]^). The CT group is defined as those who receive treatments from both an MD and a KMD. The ST group is defined as those who receive only one form of treatment from either an MD or a KMD.

For analyzing categorical data, Chi-square tests and Fisher exact tests will be used. For analyzing continuous data, Student *t*-tests, analyses of variance, and Wilcoxon signed-rank tests will be used after normal distribution has been confirmed. For analyzing missing data, the per-protocol (PP) and intention to treat (ITT) will be used. In cases of missing completely at random data and missing at random data, as defined by Rubin,^[33]^ the ITT will be used to analyze missing values using a multiple imputation^[34]^ of the PP.

For all statistical tests performed in this study, a significance level of .05 and a 2-tailed confidence interval of 95% are set up. The Stata MP version 14 (Stata Corp LLC, Texas) and SAS version 9.4 (SAS Institute Inc, Cary, North Carolina) will be used for all statistical analyses.

### 2.8 Ethics and dissemination

This study has been approved by the Institutional Review Boards of Daejeon University Dunsan Korean Medicine Hospital (DJDSKH-18-BM-06), Gil Korean Medicine Hospital, Gachon University (18-104), Dongshin University Mokpo Oriental Hospital (DSMPOS18–2), Dongguk University Bundang Oriental Hospital (DUBOH-IRB 2018-0002), Design Hospital (P01-201806-21-001), Samse Korean Medical Hospital (P01-201807-21-004), and Gangdong Mokhuri Korean Medicine Hospital (P01-201807-21-011). The protocol of this study has been registered with the Clinical Research Information Service (KCT0002827).

Based on the Declaration of Helsinki, sufficient explanation and answer will be provided and the rights and welfare of the participants are guaranteed. All data are stored in a double locked device or safe, and the corresponding author will have access to the final data. The confidentiality of personal information and anonymity will be maintained. The findings of this study will be disseminated by a report published in a peer-reviewed journal.

## 3 Discussion

In several earlier studies regarding CT, we found that the sample sizes were small and the type of KM used in CT was designated. In addition, the assessment of the effectiveness of CT mostly focused on “cooperation in the treatment”; there are few reports on the “cooperation in the diagnosis and examination.”^[17,35]^ Our study design overcomes these limitations with a relatively large sample size, no restriction on the type of KM, and by including cooperation at all clinical steps, such as examination, diagnosis, and treatment to reflect a real-world scenario.

There are a few limitations to this study protocol. First, in an observational study, researchers do not intervene in the allocation process for randomization, and there may be confounding factors. Thus, this would be a limitation to confirming a cause and effect relationship. Second, as the institutions that actively implement CT will be participating in this study, they will not be representative of the general status of CT in Korea.

Nevertheless, the clinical implications of this study are significant. By ensuring that the KM treatments are carried out without the intervention of the researchers, we can reflect a real-world clinical situation in which all forms of KM treatments, such as acupuncture, moxibustion, herbal medicine, and chuna, may be used. In addition, this study can provide basic information on complementary medicine for population health and healthcare economics.

## Author contributions

Eun Hye Hyun, NamKwen Kim, Hye-Yoon Lee, Hye Won Kim, and Hyun-Min Kim designed the study. Eun Hye Hyun drafted the manuscript. NamKwen Kim is the corresponding author, who reviewed the manuscript and will lead the study. Eun Jeong Kim, Seon Jong Kim, Yoon-Kyung Song, Young Il Kim, Woo Jin Nam, Dong Hyung Seo, and Sang-Ho Lee will serve as clinical experts and conduct the site work. All authors have read and approved of the final manuscript.

**Conceptualization:** Eun Hye Hyun, NamKwen Kim.

**Data curation:** Eun Hye Hyun.

**Formal analysis:** Eun Hye Hyun.

**Methodology:** Eun Hye Hyun, NamKwen Kim, Hye-Yoon Lee, Hye-Won Kim, Hyun-Min Kim.

**Investigation:** Eun Jung Kim, Seon Jong Kim, Yoon Gyung Song, Young Il Kim, Woo Jin Nam, Dong Hyung Seo, Sang Ho Lee.

**Project administration:** Eun Hye Hyun, NamKwen Kim.

**Supervision:** NamKwen Kim.

**Writing – original draft:** Eun Hye Hyun.

**Writing – review and editing:** NamKwen Kim.

Eun Hye Hyun orcid: 0000-0002-0011-9111
