# Clinical and cost-effectiveness of collaborative traditional Korean and Western medicine treatment for low back pain: A protocol for a prospective observational exploratory study: Erratum

**DOI:** 10.1097/MD.0000000000013202

**Published:** 2018-11-02

In the article, “Clinical and cost-effectiveness of collaborative traditional Korean and Western medicine treatment for low back pain: A protocol for a prospective observational exploratory study”,^[1]^ which appeared in Volume 97, Issue 39 of *Medicine*, Dr. YunKyung Song's name appeared incorrectly as Yoon Gyung Song.
